# Motor Control and Neural Plasticity through Interhemispheric Interactions

**DOI:** 10.1155/2012/823285

**Published:** 2012-12-26

**Authors:** Naoyuki Takeuchi, Yutaka Oouchida, Shin-Ichi Izumi

**Affiliations:** Department of Physical Medicine and Rehabilitation, Tohoku University Graduate School of Medicine, 2-1 Seiryo-Cho, Aoba-Ku, Sendai 980-8575, Japan

## Abstract

The corpus callosum, which is the largest white matter structure in the human brain, connects the 2 cerebral hemispheres. It plays a crucial role in maintaining the independent processing of the hemispheres and in integrating information between both hemispheres. The functional integrity of interhemispheric interactions can be tested electrophysiologically in humans by using transcranial magnetic stimulation, electroencephalography, and functional magnetic resonance imaging. As a brain structural imaging, diffusion tensor imaging has revealed the microstructural connectivity underlying interhemispheric interactions. Sex, age, and motor training in addition to the size of the corpus callosum influence interhemispheric interactions. Several neurological disorders change hemispheric asymmetry directly by impairing the corpus callosum. Moreover, stroke lesions and unilateral peripheral impairments such as amputation alter interhemispheric interactions indirectly. Noninvasive brain stimulation changes the interhemispheric interactions between both motor cortices. Recently, these brain stimulation techniques were applied in the clinical rehabilitation of patients with stroke by ameliorating the deteriorated modulation of interhemispheric interactions. Here, we review the interhemispheric interactions and mechanisms underlying the pathogenesis of these interactions and propose rehabilitative approaches for appropriate cortical reorganization.

## 1. Introduction

The corpus callosum, which is the largest white matter structure in the human brain, connects the homologous and nonhomologous areas of the 2 cerebral hemispheres [1, 2]. It plays a crucial role in the interhemispheric interactions that maintain independent processing and integrate information between both hemispheres [2, 3]. The functional integrity of interhemispheric interactions can be tested electrophysiologically in humans using single-pulse transcranial magnetic stimulation (TMS), double-pulse TMS, and electroencephalography [4–8]. These electrophysiological techniques were used to estimate interhemispheric transmission times (from 4 to 50 ms) [1, 3]. Structural studies using diffusion tensor imaging (DTI) have revealed the microstructural connectivity underlying interhemispheric interactions [9–12]. Moreover, functional magnetic resonance imaging (fMRI) studies have revealed interhemispheric interactions using resting-state functional and activity-dependent effective connectivity analyses [13, 14].

Research on the functions of interhemispheric interactions is based on studies of brain lateralization, which is thought to allow each hemisphere to process information without the interference of the contralateral hemisphere [15, 16]. Several studies have suggested that the speed of transcallosal conduction is limited in larger brains, which implies that the transfer and integration of information between both hemispheres through the corpus callosum require more time and energy in humans [3, 17]. Therefore, it may be more efficient to use one hemisphere and inhibit the other hemisphere during simple tasks (e.g., physical identity and face-matching tasks); this promotes intrahemispheric processing and brain lateralization [2, 18, 19].

However, processing tasks that share and integrate the information between hemispheres (e.g., dichotic word-listening task) require facilitative communication between hemispheres [20]. Even in motor tasks, the timing and accuracy of bimanual motor tasks are thought to be predominantly programmed by one of the hemispheres. To monitor the activity of the motor regions of the opposite hemisphere, sending an efference copy of the planned motor program to the opposite hemisphere through the corpus callosum allows the optimal timing of movements in both hands [21, 22]. Thus, the lateralization hypothesis can be explained by both the inhibitory and excitatory theories of interhemispheric interactions [2].

The ability to perform precisely coordinated movements using both hands is an important aspect of particular human abilities, such as tying a string, peeling a fruit with a knife, typing, and playing a musical instrument. It is now known that modulations of interhemispheric interactions are involved in the control of the unimanual and bimanual coordinations that generate the spatially and temporally precise coordinated limb movements that enable humans to perform different movements [1]. Moreover, it has been reported that interhemispheric interactions contribute to the acquisition of bimanual skills [1, 6].

Recent studies have revealed that the modulation of interhemispheric interactions relates to neural plasticity, which refers to the ability of the brain to develop new neuronal interconnections, acquire new functions, and compensate for impairments [23–25]. However, little is known about the mechanisms underlying the relation between cortical reorganization and changes in interhemispheric interaction resulting from various diseases or brain stimulation. This paper focuses on the following 4 important aspects of motor interhemispheric interactions: (1) the inhibitory and excitatory theories of interhemispheric interaction, (2) the finding that nonpathological factors can influence interhemispheric interactions, (3) the pathologies that alter interhemispheric interactions, and (4) the relation between interhemispheric interaction and neural plasticity. Assessments of interhemispheric interactions have elucidated the mechanisms underlying the physiological processes that modulate motor control and led to the formulation of interventional strategies that improve motor function after neurological disorders, which is a critical issue of clinical neurorehabilitation [25, 26]. The purposes of this paper were to provide a comprehensive overview of motor interhemispheric interactions to promote the understanding of their underlying mechanisms and to suggest approaches for appropriate neural plasticity.

## 2. The Inhibitory and Excitatory Theories of Interhemispheric Interactions

It has been estimated that the corpus callosum is the pathway through which one hemisphere can inhibit the other, thus facilitating brain lateralization. Alternatively, the corpus callosum integrates information across the cerebral hemispheres and serves an excitatory function in interhemispheric communication [2, 3, 15]. In this section, we discuss these 2 contrasting theories, the inhibitory and excitatory theories, of motor interhemispheric interaction.

### 2.1. The Inhibitory Theory

The inhibitory theory posits that the corpus callosum maintains independent processing between the hemispheres, hinders activity in the opposing hemisphere, and allows the development of hemispheric asymmetries [2]. A TMS study has demonstrated that interhemispheric inhibition from the contralateral to the ipsilateral motor cortex increases during a voluntary tonic contraction of a hand muscle [4]. This finding indicates that the voluntary activation of the motor cortex by a unimanual motor task is associated with the increased interhemispheric inhibition of the nonactive motor cortex. It is thought that this effect might work as an important mechanism for avoiding the unwanted coactivation of the nonactive motor cortex and the mirror activity of the nontask hand. This idea is further supported by the finding that the strength of the interhemispheric inhibition in healthy subjects correlates negatively with the amount of electromyographic mirror activity in the nontask hand during voluntary unilateral hand movement [27].

Handedness may be related to inhibitory interhemispheric interactions. Although it remains controversial whether interhemispheric inhibition from the dominant motor cortex differs from the nondominant motor cortex under resting condition [28–30] physiological evidence suggests an asymmetry in interhemispheric inhibition between the motor cortices during unilateral movement [5, 29]. Netz et al. demonstrated that interhemispheric inhibition from the dominant motor cortex during a voluntary tonic contraction of the dominant hand muscle was stronger than interhemispheric inhibition from the nondominant motor cortex during a voluntary tonic contraction of the nondominant hand muscle [5]. Moreover, Duque et al. showed that interhemispheric inhibition from the nondominant motor cortex was very weak at dominant hand movement onset, whereas interhemispheric inhibition from the dominant motor cortex was strong at nondominant hand movement onset [29]. These results indicate that hemispheric asymmetry promotes highly accurate control of the fine motor movements of the dominant hand by dampening the interference from the nondominant motor cortex.

### 2.2. The Excitatory Theory

The excitatory theory posits that the corpus callosum shares and integrates information between the hemispheres, resulting in greater connectivity, which decreases brain lateralization by masking the underlying hemispheric asymmetries in tasks that require interhemispheric exchange [15, 31]. This theory is supported by the disconnection syndrome, which occurs because of callosotomy. Patients with disconnection syndrome are unable to integrate information from the hemispheres, suggesting that communication between the hemispheres and the sharing of information are necessary for normal movements [15, 32, 33].

As a motor system, the excitatory interhemispheric interaction plays an important role in the adjustment of movement onset. A TMS study revealed that interhemispheric interaction from the nonactive to the active motor cortex translates from inhibitory to excitatory effects around movement onset [34]. This excitatory effect in the active motor cortex is believed to support the execution of voluntary movement. The adjustment of interhemispheric facilitation was shown not only in the primary motor cortices but also in motor-related areas. A previous study reported that the right premotor cortex also exerted an excitatory influence on the left primary motor cortex during the preparation for a movement using the dominant right hand [35]. Moreover, the excitatory interhemispheric interaction may be dependent on the cortical areas that are involved in the motor task. A recent study performed using TMS revealed that the movement-related facilitation from the right premotor to the left primary motor cortex supports the performance of antiphase bimanual movements [22]. This extent of excitatory interactions between hemispheres was positively related to the performance of antiphase bimanual movements, but not of in-phase movements. Antiphase bimanual movements are tasks that are more difficult than in-phase bimanual movements [7, 36]. The recruitment of bilateral brain regions during tasks with high levels of complexity provides evidence for an excitatory function of the corpus callosum and its ability to integrate information between the hemispheres [20]. Therefore, antiphase bimanual movements might require interhemispheric facilitation between the primary motor cortex and the premotor cortex, unlike in-phase bimanual movements.

However, the findings of interhemispheric interactions during in-phase movements support the inhibitory theory. The maximum speed of bimanual in-phase movements was the highest in subjects that exhibited weak inhibition of both homologous motor cortices [22]. Interhemispheric inhibition works to prevent mirror movements when a unimanual movement is performed, whereas interhemispheric disinhibition between homologous motor cortices may promote in-phase bimanual movements that allow the synchronous control of both hands [37–39].

These findings suggest that, depending on the motor task, the interhemispheric interactions may be inhibitory or excitatory, so that homologous muscles are adjusted [22]. This is in line with the suggestion that different channels in the corpus callosum convey either inhibitory or excitatory information between the hemispheres [31]. Moreover, this channel theory is supported by neurophysiological studies that showed that excitatory circuits through the corpus callosum share excitatory transcallosal fibers with inhibitory circuits. Interhemispheric excitatory effects result from monosynaptic connections through glutamatergic excitatory transcallosal fibers, whereas interhemispheric inhibitory effects are mediated by gamma-aminobutyric acidergic inhibitory interneurons, which are also activated by the excitatory transcallosal fibers [40, 41]. Therefore, inhibitory or excitatory interactions through interhemispheric communication can vary at different time points during the movement and according to the different cortical areas that are involved in the processing demands of the motor task or may even occur simultaneously [15].

## 3. Nonpathological Factors Can Influence Motor Interhemispheric Interactions

The degree of connectivity between the hemispheres is reflected in the size of the corpus callosum [2, 31, 42]. In addition to the size of the corpus callosum, it has been reported that age, sex, and motor training influence the interhemispheric interactions in healthy individuals. In this section, we will discuss how these factors influence motor function by altering interhemispheric interactions.

### 3.1. Age

Several studies have revealed a correlation between interhemispheric interactions and age [43–47]. The corpus callosum is not formed until 6–8 years of age [48]. In line with the anatomical findings, Mayston et al. demonstrated significant interhemispheric inhibition in adults, whereas interhemispheric inhibition was absent in children [43]. Therefore, it is thought that mirror movements occur in young children because of the immaturity of the corpus callosum, which fails to inhibit the ipsilateral motor projections or motor overflow from the active motor cortex to the nonactive motor cortex [45, 49, 50]. A developmental trend has been shown in which mirror movements decrease significantly until 6–8 years of age, which is the age range at which the myelination of the corpus callosum occurs [43, 50].

Aging also influences interhemispheric interactions. Several MRI studies have reported that aging increases the atrophy of the corpus callosum [44, 46]. Moreover, an electrophysiological study performed using TMS has revealed that aging decreases interhemispheric inhibition [47]. Therefore, in older adults, the reduction of interhemispheric inhibition might induce the reappearance of mirror movements that are observed in young children [51]. In addition to mirror movements, the age-related degeneration of the corpus callosum may alter the activity of neural recruitment. Many studies reported that healthy older adults exhibit bilateral activation of the motor cortex during a unilateral movement [52–54]. A previous report showed that recruitment of the ipsilateral motor cortex in older adults was correlated with reduced interhemispheric connectivity during a unilateral hand movement [54]. Therefore, the age-related degeneration of the corpus callosum may lead to a reduction in the hemispheric asymmetry because of the failed inhibition of the contralateral hemisphere [54, 55]. Another possible explanation for the reduction of the hemispheric asymmetry in neural activity in older adults could be that the neuronal processing in one hemisphere is reduced, requiring both hemispheres to work together to solve a given task. However, older adults exhibiting a reduction in hemispheric asymmetry during unilateral movement had poor motor performance [54]. From the point of view of the excitatory theory, the bilateral activation observed in older adults may lead to the impairment in the effective use of the excitatory interhemispheric interactions because of degeneration of the corpus callosum, resulting in a failure to compensate for the poor performance.

However, the role of the overactivation of cortices in older adults may vary according to the brain region involved in tasks. The results of previous studies supported the idea that overrecruitment of bilateral prefrontal activation compensates cognitive tasks in older adults [56, 57]. In addition to cognitive tasks, age-related increase in the activity of the supplementary motor area and left secondary somatosensory cortex was positively correlated with coordinative ability in antiphase bimanual movement [58]. The activation of bilateral hemispheres in older adults may not necessarily result exclusively from age-related dysfunction of the corpus callosum, and the increased activation observed in older adults may have positive or negative effects on performance, depending on the role played by the activated brain region in the task [54, 59, 60].

### 3.2. Sex

Several studies have reported morphological and microstructural differences in the corpus callosum between men and women. The relative size of the corpus callosum proportional to cerebral volume was larger in women compared to men [61, 62], but corpus callosum microstructural connectivity was greater in men compared to women [63, 64]. However, whether these differences in the corpus callosum observed between men and women influence functional hemispheric asymmetry remains controversial [61, 65, 66]. Therefore, in this section, we will mainly discuss the influence of female hormones on interhemispheric interactions. An effect of female hormones on the functional hemispheric asymmetry of motor control in postmenopausal women with and without female hormone therapy has been reported [67]. Similar to younger healthy subjects [68], postmenopausal women undergoing female hormone therapy exhibited pronounced functional hemispheric asymmetry during a motor task [67]. In contrast, postmenopausal control women who did not receive female hormone therapy exhibited reduced hemispheric asymmetry, similar to that observed in older adults. As mentioned previously, it is thought that a reduction of hemispheric asymmetry may partly result from the failed inhibition of the contralateral hemisphere in older adults because of an age-related dysfunction of the corpus callosum [54, 55]. Therefore, female hormones may exert positive effects on interhemispheric interactions that are related to the maintenance of independent processing between the hemispheres in the motor system [67]. Moreover, this hypothesis is consistent with the results of a TMS study that showed that young women have stronger interhemispheric inhibition compared with that in young men [69]. However, it has been reported that high estradiol and progesterone levels in young women correlate negatively with interhemispheric inhibition, as assessed using TMS [70]. In addition to interhemispheric inhibition, previous reports showed that the menstrual cycle influences motor cortical excitability [71, 72]. Although it is clear that female hormones influence interhemispheric interactions, future studies are needed to clarify the detailed mechanisms underlying the effect of female hormones on interhemispheric interactions.

### 3.3. Motor Training

As described previously, modulation of interhemispheric interactions influences human movement patterns, such as handedness. In contrast, motor training itself can change interhemispheric interactions. Changes in interhemispheric interactions mediated by motor training have been reported, especially in musical training [73–76]. Musical training is characterized by bimanual training, which includes coordinated and independent movements of both hands. Several studies have reported that musicians have more symmetrical hemispheric function than non-musicians, as assessed using evaluation methods such as speech-induced facilitation of corticospinal excitability and interhemispheric transfer time using event-related potentials for visual information [73, 74]. Moreover, it has been reported that musicians who initiated musical training early in their lives exhibit a larger corpus callosum compared with that in musicians who started learning music later in their lives and in nonmusicians [75, 76]. These results indicate that the plastic developmental changes in the corpus callosum that are caused by extensive bimanual training during childhood result in more symmetrical brains and equally efficient connections between both hemispheres because of increased interhemispheric interactions.

In addition to bimanual training, interhemispheric interactions may contribute to motor acquisitions, such as intermanual transfer, as it is well known that motor learning using one hand improves the performance of the other hand [77, 78]. A previous study using TMS revealed that unimanual sequence-specific training induces a reduction in interhemispheric inhibition of the untrained hemisphere. Moreover, this reduction in interhemispheric inhibition was correlated with an improvement in the nonspecific performance of the untrained hand [79]. Therefore, the decreased interhemispheric interaction induced by unilateral motor training may support general aspects of motor performance in the contralateral hand, rather than enhance the specific skill being learned.

In contrast to motor training, the nonused limb may also influence interhemispheric interactions. A recent study revealed that transient arm immobilization reduced the interhemispheric inhibition from the immobilized to the nonimmobilized motor cortex [80]. Moreover, this reduction in interhemispheric inhibition increased the corticospinal excitability of the nonimmobilized motor cortex when subjects were free to move the nonimmobilized arm and might result in the facilitation of the use-dependent plasticity of the nonimmobilized limb. Thus, excessive balance and imbalance between the use of both limbs modify the interhemispheric interaction and influence motor performance. However, it is illogical to think that different phenomena, such as unilateral motor training and the non-use of a limb, have a positive effect on the motor performance of the opposite limb via only a reduction in interhemispheric inhibition. Therefore, future studies are needed to identify other mechanisms, including excitatory interhemispheric interaction and/or the role of the motor-related cortices.

## 4. Pathologies Alter Interhemispheric Interactions

Studies of callosotomy or callosal lesions have provided much insight into the functions of interhemispheric interactions via the impairment of the corpus callosum [2, 15, 33]. Several neurological disorders alter interhemispheric interactions through impairment of the corpus callosum. Moreover, stroke and amputations can indirectly alter the functions of interhemispheric interactions because of imbalances between the hemispheres. In this section, we discuss the changes in the morphology and function of the corpus callosum in traumatic brain injury, multiple sclerosis, Parkinsonian syndromes, stroke, and amputation.

### 4.1. Direct Changes in Interhemispheric Interactions

Lesions of the corpus callosum are commonly detected in patients with traumatic brain injury [81–83]. Diffuse axonal injury caused by acceleration-deceleration and rotational forces is considered an important factor in the formation of a lesion of the corpus callosum [81, 82]. Electrophysiological and anatomical studies have showed that interhemispheric interactions are deteriorated after a traumatic brain injury [81–84]. A recent study using DTI revealed that the low integrity of hemispheric connections through the corpus callosum was associated with poor performance of bimanual hand movements [85].

Multiple sclerosis is an inflammatory disease that affects myelinated axons and leads to neurological and cognitive impairments. Therefore, the corpus callosum, which is the largest white matter structure in the brain, is considered a target for inflammation. Corpus callosum degeneration, which has been described frequently [86–88], can result in impaired interhemispheric communication [87], including an impairment of the interhemispheric inhibition of the contralateral motor cortex [86]. Moreover, a study using DTI showed that poor timing accuracy during a bimanual motor task was correlated with the degree of corpus callosum damage in patients with multiple sclerosis [10].

Impairments of interhemispheric inhibition detected using TMS have been reported in patients with Parkinsonian syndromes, including patients with corticobasal degeneration and progressive supranuclear palsy [89, 90]. MRI has revealed that these electrophysiological abnormalities are associated with atrophy of the corpus callosum [90, 91]. A subgroup of Parkinson's patients with mirror movements exhibited abnormally reduced interhemispheric inhibition [92]. 

Several studies using MRI reported the atrophy and reduction in microstructural connectivity of the corpus callosum in patients with schizophrenia [93, 94]. Previous longitudinal study of patients with schizophrenia suggested that the atrophy of the corpus callosum might partly result from developmental or maturational abnormalities of this structure [95]. Moreover, a reduction in the microstructural connectivity of the corpus callosum has been reported in other diseases, such as spinocerebellar ataxia types 1 and 2 (which exhibit white matter degeneration) [96] and fetal alcohol spectrum disorders (in which the white matter is possibly damaged by prenatal alcohol exposure) [97]. 

### 4.2. Indirect Changes of Interhemispheric Interactions

**Figure 1:**
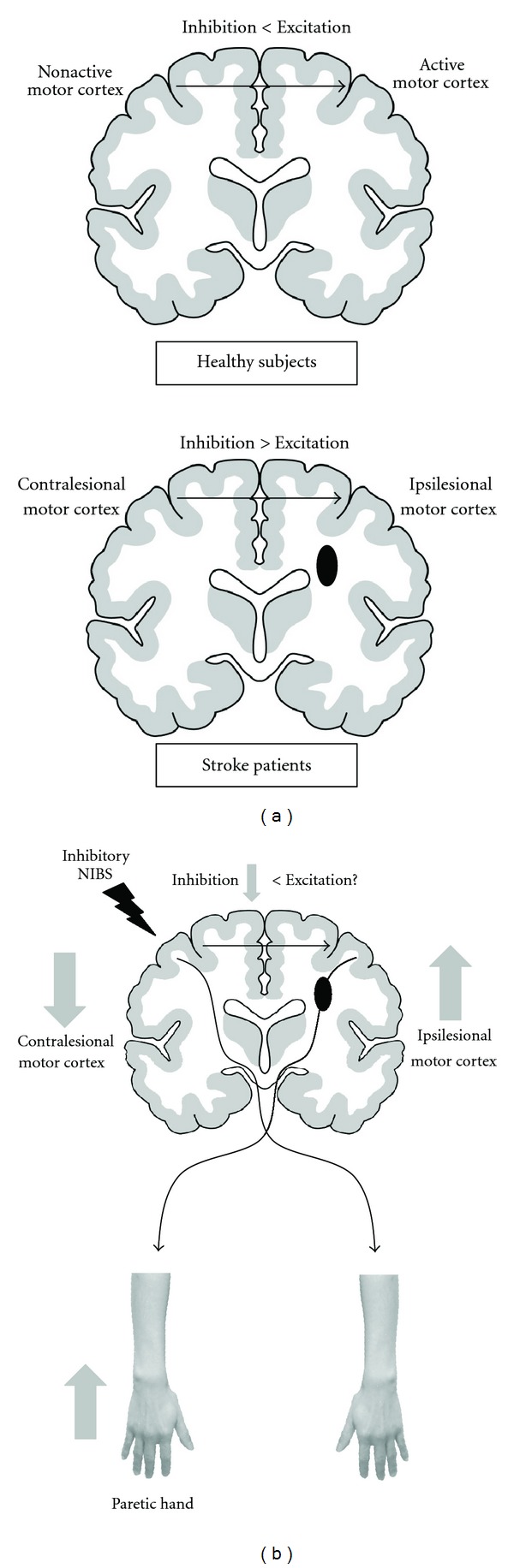
Changes in interhemispheric interaction and inhibitory noninvasive brain stimulation (NIBS) therapy in patients with subcortical stroke. (a) Mechanisms underlying the changes in interhemispheric interaction after stroke. In healthy subjects, the interhemispheric interaction changes from an inhibitory to an excitatory influence on the active motor cortex around movement onset. In contrast, stroke patients with motor deficits do not show this release from interhemispheric inhibition for the movement of the paretic hand; rather, they exhibit a persistent inhibitory influence on the ipsilesional motor cortex [34]. These pathological effects contribute to the reduced performance of the paretic hand. (b) Inhibitory NIBS over the unaffected hemisphere. Inhibitory NIBS decreases the excitability of the contralesional motor cortex and reduces the interhemispheric inhibition from the contralesional to the ipsilesional motor cortex. The excitatory interhemispheric interaction from the contralesional to the ipsilesional motor cortex might be relatively strong because of a reduced inhibitory influence. The change in interhemispheric interaction after inhibitory NIBS increases the excitability of the ipsilesional motor cortex. Facilitation of the ipsilesional motor cortex improves the motor function of the paretic hand in patients with subcortical stroke [99, 115]. However, it remains to be determined whether the excitatory interhemispheric interaction itself actually changes after inhibitory NIBS.

Several studies have reported that stroke lesions indirectly disrupt interhemispheric interactions [34, 98, 99]. TMS studies have showed that interhemispheric inhibition persisted from the unaffected to the affected hemisphere around the onset of the movement of the paretic hand in stroke patients, whereas the interhemispheric interaction in healthy controls changed from inhibitory to excitatory influence on the active motor cortex closer to the time of movement onset [34, 98]. This abnormal adjustment of interhemispheric inhibition correlates with motor function deficits, strongly suggesting that altered interhemispheric interactions can result in motor deficits in patients with stroke [34, 98] (Figure 1(a)). The increased excitability in the unaffected hemisphere because of an imbalance in both hemispheres and excessive use of the nonparetic side after stroke, resulting in overactive excitability in the unaffected hemisphere that strongly inhibits the affected hemisphere through the corpus callosum, is a mechanism that could possibly explain this observation [25, 34]. Moreover, an fMRI study using an activity-dependent connectivity analysis also reported that the amount of inhibitory influence from the contralesional to the ipsilesional motor cortex during the movement of the paretic hand was negatively correlated with the motor function of the paretic hand in patients with subcortical stroke [100]. Thus, the issue of how interhemispheric interactions affect motor performance is highly relevant to the assessment of motor recovery after stroke [101, 102]. However, a relation between excessive interhemispheric inhibition from the contralesional motor cortex and motor impairment has been reported mainly in patients with chronic subcortical stroke and during movement. The interhemispheric interaction may vary depending on the stage of the stroke, the site of the lesion, and movement conditions [14, 103, 104]. In contrast to the studies that the excessive interhemispheric interaction had a negative effect of motor recovery [34, 98, 100], fMRI study reported that the resting-state functional connectivity between both hemispheres became strong with motor recovery in patients with subcortical stroke [13]. Therefore, longitudinal neuroimaging and electrophysiological studies must be performed to demonstrate the dynamic change in interhemispheric interaction between both hemispheres during the process of functional recovery [14, 103].

**Figure 2:**
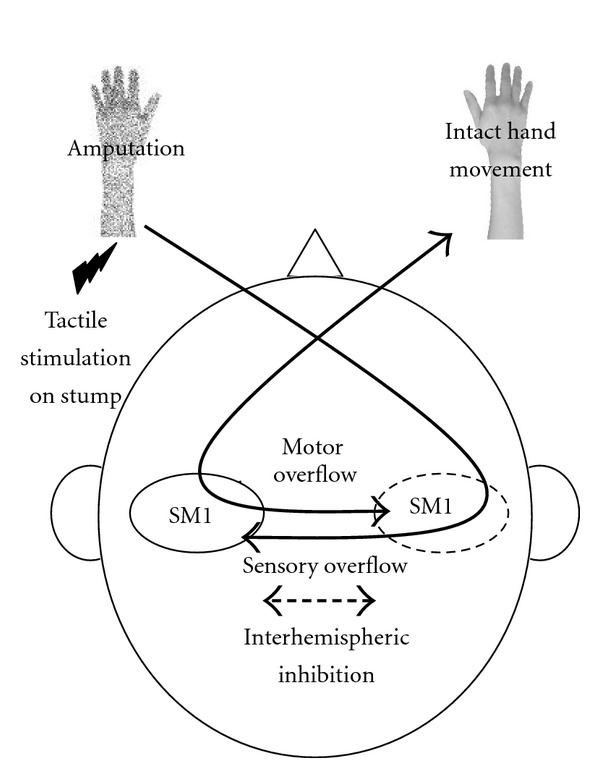
Amputation alters the interhemispheric interactions through the corpus callosum and induces bilateral neural activity. After amputation, reorganization of the deafferented sensorimotor cortex (SM1) occurs due to the absence of an afferent input from the missing hand. This change leads to an imbalance between the hemispheres in patients with amputations. Moreover, experience-dependent changes in representation by overuse of the intact hand increase this imbalance between the hemispheres. The imbalance between the hemispheres alters the interhemispheric interactions through the corpus callosum. In particular, the reduced interhemispheric inhibition observed in patients with amputations induces the neural activation of both hemispheres due to the failed inhibition of the opposite hemisphere. When tactile stimulation is delivered to the stump of the amputated limb, the overflow of the afferent information induces the activation of the nondeafferented SM1. In addition to the sensory system, the motor overflow increases the activity of the deafferented SM1 during the movement of the intact hand.

In addition to stroke, recent studies revealed that indirect changes in interhemispheric interactions through the corpus callosum occur after changes in peripheral organs, such as limb amputation [105, 106]. This change in interhemispheric interaction may reflect the interhemispheric imbalance induced by the reorganization of the deafferented sensorimotor cortex after amputation and/or experience-dependent changes in the representation of the overuse of the intact limb [107, 108]. Recently, Simões et al. showed that patients with amputations had decreased microstructural connectivity of the corpus callosum compared with that in healthy volunteers [105]. A previous study with DTI demonstrated that the microstructural connectivity of the corpus callosum positively correlated with the degree of interhemispheric inhibition in healthy volunteers [109]. Therefore, the reduced connectivity of the corpus callosum observed in patients with amputations may induce bilateral neural activation, which is possibly due to the failed inhibition of the opposite hemisphere [105, 106, 108] (Figure 2). In fact, previous studies revealed the presence of reduced hemispheric asymmetry in patients in whom an intact hand movement increased the activity of the deafferented sensorimotor cortex [108, 110, 111]. A reduction in hemispheric asymmetry on sensory system was also shown in a recent fMRI study performed in patients with amputations. In that study, the somatosensory areas on both sides were activated by stimulation of the stump area on the amputated limb [106]. Thus, amputation induced a reduction in hemispheric asymmetry in both the sensory and motor systems via a change in interhemispheric interaction. Although future studies must be performed to identify methods that can restore deteriorated interhemispheric interaction after amputation, a recent study reported that neurally driven prosthesis training normalizes abnormal electroencephalography coherence between both sensorimotor cortices [112]. Therefore, therapies such as prosthesis and mirror therapy can induce the reorganization of the deafferented sensorimotor cortex via visual and somatosensory feedback [113, 114], which might normalize the interhemispheric interaction after amputation.

## 5. Relation between Interhemispheric Interactions and Changes in Neural**  **Plasticity

It has been reported that several techniques alter interhemispheric interactions. In particular, noninvasive brain stimulation (NIBS), which can modulate cortical excitability, may enhance neural plasticity by altering interhemispheric interactions. Moreover, paired associative stimulation of the homologous motor cortices using TMS induces a neural plasticity that is dependent on Hebbian mechanisms through interhemispheric interactions. In this section, we discuss the neural plasticity that is induced by changes in interhemispheric interactions.

### 5.1. Brain Stimulation Alters Interhemispheric Interactions

Repetitive TMS and transcranial direct current stimulation are NIBS techniques that can alter the excitability of the human cortex for several minutes [115]. In particular, it has been reported that inhibitory NIBS over the motor cortex decreases the excitability of the stimulated motor cortex, which leads to a reduction in the interhemispheric inhibition from the stimulated motor cortex to the nonstimulated motor cortex [116, 117]. Moreover, the reduction in interhemispheric inhibition from the stimulated to the non-stimulated motor cortex increases the excitability of the non-stimulated motor cortex. In turn, the increased excitability of the non-stimulated motor cortex induces improvements in motor performance on the ipsilateral side [118, 119]. In addition, the increased motor cortical excitability induced by inhibitory NIBS enhances the effects of motor training on the ipsilateral side [120, 121], as the increase in excitability in the motor cortex appears to be a necessity for motor learning [122, 123].

A recent study reported that paired associative stimulation of the homologous motor cortices using TMS is a new interventional protocol that induces an increase in excitability in the conditioned motor cortex [124]. The paired associative stimulation of the 2 motor cortices induces highly synchronized action potentials in corticospinal output neurons in the 2 motor cortices and improves the motor function of the hand that is innervated by the conditioned motor cortex. The effect of paired associative stimulation results from the reduction of interhemispheric inhibition to the homologous conditioned motor cortex [124]. Moreover, its effect is strongly dependent on the timing of the delivery of the stimulus pairs (8 ms), corresponding to the interval time between the double-pulse TMS that induces the interhemispheric inhibition [4]. It is thought that paired associative stimulation induces a neural plasticity that is dependent on the Hebbian learning rule via which synapses increase their efficacy if the synapse consistently assists the postsynaptic target neuron in the generation of action potentials [125].

### 5.2. Motor Stroke Therapy via Interhemispheric Interaction Modulation

As mentioned previously, excessive interhemispheric inhibition from the unaffected hemisphere deteriorates the motor function of the paretic hand in patients with stroke. Therefore, improvement of the motor deficits of these patients may be achieved by decreasing the excitability of the unaffected hemisphere using NIBS [101, 102]. In fact, it has been reported that inhibitory NIBS over the unaffected hemisphere in patients with stroke decreases the interhemispheric inhibition from the unaffected hemisphere to the affected hemisphere and increases the excitability of the affected hemisphere, resulting in facilitated motor learning and motor recovery in the paretic hand [99, 115] (Figure 1(b)). A recent study also suggested that inhibitory NIBS over the contralesional motor cortex might influence the ability of the ipsilesional motor cortex to undergo plastic modifications by preparing the cortical ground for successful use-dependent plasticity in stroke patients [126].

Although it has been reported that inhibitory NIBS over the unaffected hemisphere facilitates motor recovery during the acute stage of stroke [127, 128], a recent study showed that inhibitory NIBS did not facilitate motor recovery in patients with stroke in the acute stage [129]. This implies that the interhemispheric inhibition from the contralesional to the ipsilesional motor cortex does not necessarily correlate with motor impairment in all patients with stroke. Moreover, Lotze et al. have shown that disrupting the contralesional motor cortex via TMS may cause deterioration of the complex motor performance of the paretic hand in patients with chronic stroke with internal capsule infarcts [130]. Therefore, inhibitory NIBS delivered over the contralesional motor cortex might be associated with a risk of deteriorating complex movements in some patients with stroke. Furthermore, it has been noted that inhibitory NIBS reduces the interhemispheric inhibition that controls bimanual movement [131, 132]. In fact, recent studies reported that inhibitory repetitive TMS over the unaffected hemisphere transiently deteriorated performance in the antiphase bimanual tapping task in patients with stroke [133, 134]. Therefore, it should be noted that inhibitory NIBS is associated with a risk of deteriorating some motor functions by altering the motor network system [103, 135]. 

## 6. Conclusion

This paper focused on the mechanisms underlying motor control and neural plasticity that relate to interhemispheric interactions to suggest approaches for appropriate cortical reorganization. Inhibitory or excitatory interactions that occur via interhemispheric communication may vary depending on the different time points during the movement and different cortical areas that are involved in the processing demands of the motor task. The age-related degeneration of the corpus callosum may induce the engagement of both hemispheres partly because of the failed inhibition of the contralateral hemisphere. Female hormones may exert positive effects on the interhemispheric communication that is related to maintaining independent processing between the hemispheres in the motor system. Plastic developmental changes that are caused by extensive bimanual training during childhood result in more symmetrical brains and equally efficient connections between the hemispheres. Several neurological disorders, such as traumatic brain injury, multiple sclerosis, and Parkinsonian syndromes, directly alter interhemispheric interactions by impairing the corpus callosum. Stroke lesions indirectly disrupt interhemispheric inhibition, which is highly relevant to the research on motor recovery after stroke. In addition, amputations may indirectly alter interhemispheric interactions between sensorimotor cortices. Inhibitory NIBS reduces the interhemispheric inhibition from the stimulated motor cortex to the non-stimulated motor cortex. The paired associative stimulation of the homologous motor cortices using TMS induces a neural plasticity that is dependent on Hebbian mechanisms that occur via interhemispheric interactions. Inhibitory NIBS over the unaffected hemisphere in patients with stroke can improve the motor function of the paretic hand by reducing the interhemispheric inhibition from the unaffected hemisphere to the affected hemisphere. However, it should be noted that inhibitory NIBS might worsen bimanual movements by reducing the interhemispheric inhibition that controls them. Assessments of interhemispheric interactions have provided information on the mechanisms underlying the physiological processes involved in motor control and have allowed the formulation of interventional strategies that can improve motor function in neurological disorders, which is a critical issue in clinical neurorehabilitation.
